# Relation of Leptin, Ghrelin and Inflammatory Cytokines with Body Mass Index in Pulmonary Tuberculosis Patients with and without Type 2 Diabetes Mellitus

**DOI:** 10.1371/journal.pone.0080122

**Published:** 2013-11-08

**Authors:** Ying Zheng, Aiguo Ma, Qiuzhen Wang, Xiuxia Han, Jing Cai, Evert G. Schouten, Frans J. Kok, Yunchun Li

**Affiliations:** 1 Institute of Human Nutrition, Medical College of Qingdao University, Qingdao, P. R. China; 2 Division of Human Nutrition, Wageningen University, Wageningen, The Netherlands; Boston University, United States of America

## Abstract

**Background:**

Pulmonary tuberculosis (TB) patients often suffer from anorexia and poor nutrition, causing weight loss. The peptide hormones leptin and its counterpart ghrelin, acting in the regulation of food intake and fat utilization, play an important role in nutritional balance. This study aimed to investigate the association of blood concentrations of leptin, ghrelin and inflammatory cytokines with body mass index (BMI) in TB patients with and without type 2 diabetes mellitus (T2DM).

**Methods:**

BMI, biochemical parameters and plasma levels of leptin, ghrelin and inflammatory cytokines were measured before the start of treatment in 27 incident TB patients with T2DM, 21 TB patients and 23 healthy subjects enrolled in this study.

**Results:**

The levels of leptin were significantly higher in TB patients (35.2±19.1 ng/ml) than TB+T2DM (12.6±6.1 ng/ml) and control (16.1±11.1 ng/ml) groups. The level of ghrelin was significantly lower in TB (119.9±46.1 pg/ml) and non-significantly lower in TB+T2DM (127.7±38.6 pg/ml) groups than control (191.6±86.5 pg/ml) group. The levels of TNF-α were higher, while IFN-γ and IL-6 levels were lower in patients than in the control group. Leptin showed a negative correlation with BMI in TB (r=-0.622, *p*<0.05) and TB+T2DM (r= -0.654, *p*<0.05) groups, but a positive correlation with BMI in the control group (r=0.521, *p*<0.05). Contrary ghrelin showed a positive correlation with BMI in TB (r=0.695, *p*<0.05) and TB+T2DM (r= 0.199, *p*>0.05) groups, but negative correlation with BMI in the control (r=-0.693, *p*<0.05) group. Inflammatory cytokines were poorly correlated with BMI in this study. Only IFN-γ showed a significant negative correlation with BMI in the control group (r=-0.545, *p*<0.05).

**Conclusions:**

This study may suggest that possible abnormalities in ghrelin and leptin regulation (high levels of leptin and low levels of ghrelin) may be associated with low BMI and may account for the poor nutrition associated with TB and TB+T2DM.

## Introduction

Pulmonary tuberculosis (TB) is a major cause of mortality around the world, nearly one-third of the world’s population is infected, and 8~12 million people become newly infected each year [1]. TB incidence is influenced by several social and economic factors, such as poverty or poor nutrition [2], as well as by other diseases, such as diabetes mellitus (DM). Many studies now show that DM may be associated with an increased risk (almost triple) of developing active TB [3-5], and TB patients who also have diabetes may have higher rates of treatment failure and death [6,7]. China has the second highest rate of TB morbidity in the world and DM rates are reaching epidemic proportions [8]. This harmful synergy of TB and DM has led public health systems in China to attempt to tackle the two diseases concurrently. A study was initiated in which active TB patients in poverty zones are screened for DM and the effect of a diet and lifestyle intervention is evaluated [9].

Poor nutrition represented by wasting and anorexia, is a prominent feature of both TB and DM, being a hypercatabolic state, characterised by accelerated protein degradation and muscle wasting, resulting in weight loss, and deteriorating clinical functions with resultant poor prognosis [4]. The appetite-related hormones, leptin and ghrelin, may be new candidate causes of TB-associated malnutrition [10-13]. Leptin is a protein hormone of 167 amino acids. Its main effect relates to energy exhaustion and control of food intake, implicated as an anorexigenic factor that reduces appetite [14].The connection of leptin to adipose tissue emphasizes the endocrine function of that compartment. Conversely, ghrelin is a 28-amino-acid peptide, which is produced by the stomach and has recently attracted interest as a novel anti-catabolic and orexigenic factor, that is increased in anorexic conditions and stimulates appetite [15]. Many studies have also revealed that both leptin and ghrelin are immune system regulators in addition to their effect on food intake [15,16]. Plasma levels of leptin and ghrelin can be altered in disease states associated with anorexia [10-13]. However, previous data regarding leptin levels in TB patients are conflicting. One study has shown that pretreatment plasma leptin levels were lower in TB patients than in healthy controls and there was a strong positive correlation between leptin concentration and body mass index (BMI) in both the control and patient group [11]. Serum leptin level was found to be higher in TB patients than controls in other studies [13,17] in which leptin levels were positively related to BMI only in the control group. Ghrelin has not been widely studied in patients with TB [18-20], let alone patients with TB+DM. There may be increased activation of the inflammatory system and alterations of the immune system in TB and TB+T2DM [21-23]. Leptin and ghrelin secretion and their circulating levels are effected by diet, adiposity, energy balance, and hormonal factors together with many intrinsic adiposity factors and cytokines [24]. Tumor necrosis factor-alpha (TNF-α) shows antimycobacterial activity and promotes granuloma formation in TB patients [25]. The increase of TNF-α· may cause anorexia and consequent weight loss in TB patients [26]. Interferon-gamma (IFN-γ), a Th1-type cytokine, is known to be a key cytokine in the host immune response to tuberculosis infection. If IFN-γ cannot be produced or cannot exert its effects, TB infection is more severe and often fatal [25].Some studies showed there were negative correlations between inflammatory mediators like CRP, IL-1 and TNF-α with BMI in patients with active lung tuberculosis [11,17,27]. However, evidence for a link between the inflammatory response and malnutrition is still equivocal and Incomplete [22,28]. Whether weight loss in tuberculosis is probably due to over release of cytokines remains unknown. 

This study was undertaken to investigate whether the plasma levels of leptin, ghrelin and inflammatory cytokines are associated with BMI (reflecting nutritional status) in TB patients with and without type 2 diabetes (T2DM).

## Materials and Methods

### Ethics Statement

 Permission from the ethics committee of the affiliated hospital of Medical School of Qingdao University was obtained before the study. And study was conducted according to the principles outlined in the Declaration of Helsinki. Study subjects were informed, each submitted written informed consent before the study.

### Subjects

27 patients with TB +T2DM from the Chest Hospital of Qingdao were enrolled prospectively in this cross sectional study. TB+T2DM patients had positive sputum culture of mycobacterium tuberculosis and positive chest X ray, also had a fasting plasma glucose ≥7.0 mmol/l (mM) or a random blood sugar >11.1 mM 21 TB patients (also from chest hospital of Qingdao) and 23 healthy subjects from medical center of the affiliated hospital of Qingdao medical college both with similar age and gender distribution were enrolled. Patients with type 1 diabetes, miliary TB, non-tuberculous mycobacteria (NTM), or human immunodeficiency virus co-infection were excluded. Patients and control subjects who had any other serious concomitant diseases or had been previously treated with anti-TB drugs were also excluded. Informed consent was obtained from all subjects.

### Measurements

A blood sample was collected before any treatment had been given via a venous catheter into a Heparin Sodium tube and non- anticoagulation tube (5ml respectively), between 7 and 8 AM after overnight fasting. After centrifugation of the heparin sodium tube, plasma was stored at -80°C. Samples with non-anticoagulation tubes, were water bathed for 20~30min at 37°C, and then centrifuged at 1580g for 5 min. All biochemical analyses (including fasting plasma glucose, hemoglobin, lipids, hepatic function parameter) in non-anticoagulation tubes were performed on 7600-210 automatic biochemistry analyzer (HITACHI, Inc, Japan), using Synchron reagents provided by leadmanbio, Beijing. Blood samples collected in the Heparin Sodium tubes for the following assesments: plasma levels of leptin (eBioscience; BMS2039INST, USA), total ghrelin (Millipore, EZGRA-88K, USA), TNF-α (Peprotech; 900-M25, USA), interleukin-6 (IL-6) (Peprotech; 900-M16, USA) and IFN-γ (Peprotech; 900-M27, USA) were determined using enzyme-linked immuno sorbent assay. Subjects were weighed barefoot with minimum clothing using an electronic weighing scale, body weight was recorded to the nearest 0.1 kg. Height was measured to the nearest 0.1 cm using stadiometer, the BMI was calculated as Weight (kg)/ height^2^ (m).

### Statistical analysis

The sample size was estimated with a two-sided alpha of 5% and a power of 80%. Data were tested for normal distribution using the Kolmogorov–Smirnov test. If the distribution appeared nonnormal, the continuous variables were transformed by calculating the log_10_ prior to using parametric test statistics. Results are expressed as mean±SD for normally distributed data and geometric means for log_10_ transformed data. Statistical testing for transformed variables were carried out by ANOVA if the data were normally distributed, and by non-parametric tests if the data were non-normally distributed. Correlations between transformed variables were estimated using pearson correlation coefﬁcient. Multivariate linear regression analysis was carried out to evaluate the relationship between the parameters. SPSS 11.5 software was used for all statistical analyses and a *p*-value <0.05 was deemed statistically significant.

## Results

The anthropometric and biochemical characteristics of the 3 groups are shown in Table 1. TB (23.0±4.3 kg/m^2^) and particularly TB+T2DM (22.2±3.5 kg/m^2^) groups had lower levels of BMI than the control group (24.8±3.6 kg/m^2^). TB+T2DM showed a higher level of serum glucose than TB and control groups (10.6±4.4 versus 5.1±0.5 mM, 10.6±4.4 versus 5.2±0.5 mM, respectively, *p*<0.05). The control group had higher level of HDL-cholesterol, proteins and uric acid than patients groups (*p*<0.05). 

**Table 1:** Characteristics of the study subjects (N= 71).

	TB	TB+ T2DM	Control
N(male/female)	21(17/4)	27(23/4)	23(20/3)
Age(years)	50.2±10.9	52.9±10.3	49.4±15.6
BMI(kg/m^2^)	23.0±4.3	22.2±3.5 ^c^	24.8±3.6
Glucose(mM)	5.1±0.5	10.6±4.4 ^a, c^	5.2±0.5
Cholesterol(mM)	5.9±7.6	4.4±1.3	4.7±0.8
Triglycerides(mM)	1.1±0.2	1.12±0.5	1.1±0.3
VLDL-cholesterol(mM)	2.6±0.7	2.3±0.7	2.2±0.3
HDL-cholesterol(mM)	1.1±0.3 ^b^	1.2±0.3 ^c^	1.3±0.3
Total protein(mM)	62.6±7.5 ^b^	63.9±7.6 ^c^	75.9±4.1
Albumin(mM)	39.1±7.0 ^b^	37.9±5.4 ^c^	43.8±2.4

Results are expressed as mean±SD.

Abbreviations: BMI, body mass index; VLDL, very low-density lipoprotein; HDL, high-density lipoprotein.

a Compared TB with TB+T2DM，*p*<0.05; b: Compared TB with Control，*p*<0.05 c: Compared TB+T2DM with Control, *p*<0.05.

The levels of peptide hormones and inflammatory cytokines are shown in Table 2. The level of leptin was significantly higher in TB patients than in TB+T2DM and control groups (35.2±19.1 versus 12.6±6.1 ng/ml, 35.2±19.1 versus16.1±11.1 ng/ml respectively, *p*<0.05), while the level of ghrelin was lower in TB (119.9±46.1 pg/ml) and TB+T2DM (127.7±38.6 pg/ml) groups compared with control group (191.6±86.5 pg/ml). Levels of TNF-α were higher in TB and TB+T2DM compared with control group (486.9±30.4 versus 340.9±23.6 pg/ml, *p*>0.05; 616.0±24.9 versus 340.9±23.6 pg/ml, *p*<0.05). Levels of IL-6 were higher in control group but the differences were not significant. Levels of IFN-γ were lower in TB and TB+T2DM compared with control group (2278.7±929.2 versus 4020.7±838.5 pg/ml, *p*<0.05; 3501.2±488.3 versus 4020.7±838.5 pg/ml, *p*>0.05).

**Table 2:** Circulating levels of appetite-related hormones and inflammatory cytokines in patients and healthy subjects (N= 71).

	TB	TB+ T2DM	Control
N	21	27	23
Leptin(ng/ml)	35.2±19.1 ^a ,b^	12.6±6.1	16.1±11.1
Ghrelin(pg/ml)	119.9±46.1 ^b^	127.7±38.6	191.6±86.5
TNF-α(pg/ml)	486.9±30.4	616.0±24.9 ^c^	340.9±23.6
LogIL-6(pg/ml)	24.2(19.6-28.8)	26.8(22.8-30.8)	29.9(25.0-30.8)
IFN-γ(pg/ml)	2278.7±929.2 ^b^	3501.2±488.3	4020.7±838.5

Values are mean±SD, and geometric mean (95% CI) for Log IL-6.

Abbreviations: IL-6, interleukin-6; TNF-α, denotes tissue necrosis factor-α; IFN-γ,,interferon-γ.

a Compared TB with TB+T2DM，*p*<0.05; b: Compared TB with Control，*p*<0.05 c: Compared TB+T2DM with Control, *p*<0.05.

Table 3 shows the correlation of appetite-related hormones and inflammatory cytokines with BMI among the 3 groups. Leptin showed a negative correlation with BMI in TB (r=-0.622, *p*<0.05) and TB+T2DM (r= -0.654, *p*<0.05) groups, but positive correlation with BMI in control group (r=0.521, *p*<0.05). Ghrelin showed positive correlation with BMI in the TB (r=0.695, *p*<0.05) and TB+T2DM (r= 0.199, *p*>0.05) groups, but negative correlation with BMI in control group (r=-0.693, *p*<0.05). There were positive nonsignificant correlations for all 3 cytokines in the TB group, negative nonsignificant correlations for TNF-α and IFN-γ and a positive nonsignificant correlation for IL-6 in the TB+T2DM group. In the control group, all correlations were negative and nonsignificant, except for IFN-γ (r=-0.545, *p*<0.05).

**Table 3:** Correlation of characteristics with BMI among different groups.

	TB	TB+ T2DM	Control
N	21	27	23
Leptin	-0.622*	-0.654*	0.521*
Ghrelin	0.695*	0.199	-0.693*
TNF-α	0.163	-0.085	-0.350
Log10IL-6	0.293	0.364	-0.313
IFN-γ	0.205	-0.211	-0.545*

Abbreviations: IL-6, interleukin-6; TNF-α, denotes tissue necrosis factor-α; IFN-γ, interferon-γ * *p*<0.05.

Multivariate linear regression of BMI as dependent variable with age, sex, appetite-related hormones and inflammatory cytokines in the three groups are showen in Table 4. Leptin was significantly inversely associated with BMI in the TB (*p*=0.009) and TB+T2DM (*p*=0.012) groups, but positively in the control group (*p*=0.436). Ghrelin had a significant positive association with BMI in the TB (*p*=0.026) and TB+T2DM (*p*=0.049) groups, but an inverse association in the control group (*p*=0.233). There were no important associations between BMI and age, sex, inflammatory cytokines in 3 groups respectively. Leptin was the major factor affecting BMI in the TB group, while ghrelin was the major factor in the TB+T2DM group. 

**Table 4:** Multivariate linear regression of BMI with age, sex, appetite-related hormones and inflammatory cytokines in different groups.

	TB		TB+ T2DM		Control	
	B Coefficients	*P*	B Coefficients	*P*	B Coefficients	*P*
Age(year)	-0.025	0.731	-0.006	0.916	-0.007	0.913
Sex	2.234	0.218	0.236	0.913	0.018	0.990
Leptin(ng/ml)	-3.873	0.009	-0.164	0.012	0.037	0.436
Ghrelinpg/ml)	1.341	0.026	0.531	0.049	-0.330	0.233
TNF-α(pg/ml)	0.00	0.874	0.001	0.791	0.001	0.891
Log10IL-6(pg/ml)	0.082	0.299	0.109	0.086	-0.065	0.278
IFN-γ(pg/ml)	0.113	0.156	0.002	0.976	-0.110	0.174

Dependent Variable: BMI. Abbreviations: IL-6, interleukin-6; TNF-α, denotes tissue necrosis factor-α; IFN-γ,interferon-γ.

## Discussion

 In our study, we found TB patients with or without T2DM had a worse nutritional status (lower BMI and plasma protein levels) than controls. Our results demonstrate that plasma leptin levels were significantly higher in the TB group compared to the TB+T2DM and control groups, whereas levels of plasma ghrelin were lower in patients than in the control group. The association with BMI was negative for leptin and positive for ghrelin in the patients whereas these associations were opposite in control group. Levels of TNF-α were higher and IFN-γ were lower in the patients compared with the controls. However, in mulivariate analysis, the association of leptin and ghrelin with BMI was not explained by the cytokines.

 Our study examined not only TB and healthy subjects, but TB+T2DM patients as well, a group which has rarely received attention until now. This relates to the special situation in China where TB and DM constitute a double-burden. Patients in our study were not yet treated by anti-tuberculosis drugs. Limitations were the relatively small sample size, and the small number females within group, so the sex effect on related hormones and inflammatory cytokines could not be investigated. We have measured total ghrelin levels in the plasma, which contains both “active” and “inactive” forms. However, it has been reported that total ghrelin levels can reflect the level of the active form in the plasma [29,30]. In this study, we chose BMI as a correlate of body fat. The hospital in our area could not afford equipment to measure the percent body fat. Otherwise, body fat estimated from skinfold thickness is dependent on accurate measurements at the right sites (biceps, triceps, subscapular, and suprailiac regions), this requires skillful staff to carry out the work. However, patients in our study were mainly from counties and villages in poor rural areas, and although skinfold thickness was measured, it was difficult to train local staffs to perform standardized measurements for percent body fat measurement. According to many reports, BMI could effectively predict the percentage of body fat. Suchanek P et al [31] aimed to compare estimates of body fat content, like BMI, body adiposity index (BAI), waist-hip ratio (WHR), with respect to their ability to predict the percentage of body fat. They found BMI index was the better universally valid index to predict the percentage of body fat than BAI index and WHR index. Also others reported a good correlation of BMI and percentage of body fat especially in Asian. The correlation coefficient (R) could go up to 0.90 [32]. Researchers in China also made many investigations to evaluate the possibilities to use BMI as predictor of body fat status and diseases caused by obesity [33–35]. With respect to difference according to race, Asian people have less muscle and skeleton tissue than westerns with the same BMI, and the percentage of body fat is higher. So given the quality of the skinfold thickness measurements in our study and based on the reports of correlation between BMI and body fat in Asians, we prefer to use BMI as a correlate of body fat applicable to individuals in China. 

TNF-α induces fever and weight loss, which are prominent symptoms of TB. It has been shown to be associated with both protection and pathogenesis in mycobacterial infections [36]. Raised concentrations of TNF-α may underly much of the metabolic clustering due to diabetes mellitus [37]. In our study, the level of TNF-α was higher in TB patients, especially those with T2DM. This finding may suggest that mycobacterium tuberculosis (MTB), mainly multiplying in macrophages，stimulates TNF-α release, especially in the presence of high glucose concentrations. IFN-γ as one of the typical Th1 cytokines is a key cytokine in the host immune response to tuberculosis infection. We found that TB patients with or without T2DM had lower levels of IFN-γ than controls like others reported [38], which may indicate that they have a weaker defense against tuberculosis infection.

Leptin is mainly synthesized by adipose tissue. But under inflammatory conditions, one study suggested a ‘‘cytokine–leptin hypothesis’’ implying that higher multiple cytokine levels are correlated with increased leptin levels [39]. In the TB patient group, we found higher levels of TNF-α and leptin compared to the control group. However, the TB+T2DM group had higher levels of TNF-α in combination with lower leptin levels compared to the control group in our study. Experimental studies showed that the leptin levels in patients with type 2 diabetes are diverse and seem to be related to the duration of the disease. In subjects with poorly controlled type 2 diabetes who lost weight rapidly, with lower levels of BMI and leptin, levels of TNF-α were reported to be higher than in controls [40,41]. So possibly in patients with type 2 diabetes, leptin and TNF-α status can not be explained by the ‘‘cytokine–leptin hypothesis’’. Therefore in TB patients with T2DM, the pathogenesis of wasting may be different. To our knowledge there have been no reports in the literature on this. Maybe there is a more complicated mechanism at work when TB and T2DM are combined. Leptin may be an important factor involved in the mechanism behind wasting, because both in TB patients with and without T2DM, leptin showed a negative correlation with BMI. Two articles [18,20] found that ghrelin in TB patients is elevated compared to controls, and correlates negatively with BMI [20]. But Kim et al [19] found that plasma ghrelin levels were significantly lower in malnourished patients than in well nourished patients with TB before treatment. In correlation analysis, ghrelin levels were negatively correlated to the malnutrition score. Our results were similar showing a positive correlation of ghrelin with BMI in TB and TB+T2DM groups as Kim et al [19]. 

 Interestingly, the correlation of leptin and ghrelin with BMI in the patient groups is contrary to what is observed in the healthy control group where it is correlated with the amount of fat tissue.as reported in the literature [13,17]. In TB patients high leptin levels may be a cause of the low BMI instead of being a consequence of the amount of fat tissue. Inflammatory cytokines were poorly correlated with BMI in our patients, although there was a negative association of leptin and TNF-α with BMI. Cakir B et al [17] have found increased and correlated levels of TNF-α and leptin in tuberculosis patients, and his interpretation was that the elevated leptin level leads to weight loss, and through that may contribute to the inflammatory process. However, Kim JH et al [19] found higher levels of TNF-α in the TB group, that were poorly correlated with weight loss, whereas leptin levels were positively correlated with TNF-α, although not statistically significant. Combined with our findings, higher levels of leptin and TNF-α may indicate that although TNF-α may correlate with leptin, it is not directly responsible for the lower BMI. Although some reports have shown that plasma TNF-α levels are associated with weight loss in TB, we were unable to demonstrate this. Although high inflammatory status maybe primarily responsible for low BMI in patients with TB, low BMI in TB cannot be explained by the investigated inflammatory cytokines only [21,42]. This may suggest that externalization of leptin and ghrelin in these patients is not determined by nutritional status (amount of fat tissue), but in fact is a causal factor in the development of low BMI. The effect of leptin and ghrelin on BMI seems to be largely independent of the investigated cytokines. Further studies are needed to investigate the complex mechanisms. 

## Conclusions

This study provides novel evidence of status and relationship between appetite-related hormones, inflammatory cytokines and BMI in TB patients with or without T2DM. We conclude that possible abnormalities in leptin and ghrelin regulation may be associated with the development of poor nutrition (low BMI) during the inflammatory response in TB patients with or without T2DM. TB patients with T2DM may have more complex and different pathogenesis compared to TB patients only. Given the complexity of the interaction of appetite-related hormones and cytokines, further studies are needed to clarify this issue and underlying mechanisms.

## References

[1] World Health Organization (2011) Guidelines for the programmatic management of drug-resistant tuberculosis World Health Organization Document 6: 1–33 23844450

[2] Lönnroth K, Jaramillo E, Williams BG, Dye C, Raviglione M (2009) Drivers of tuberculosis epidemics: the role of risk factors and social determinants. Soc Sci Med 68: 2240–2246. doi:10.1016/j.socscimed.2009.03.041. PubMed: 19394122. 19394122

[3] Jeon CY, Murray MB (2008) Diabetes mellitus increases the risk of active tuberculosis: a systematic review of 13 observational studies. PLOS Med 5: 152. doi:10.1371/journal.pmed.0050152. PubMed: 18630984. PMC2459204 18630984

[4] Stevenson CR, Critchley JA, Forouhi NG, Roglic G, Williams BG et al. (2007) Diabetes and the risk of tuberculosis: a neglected threat to public health? Chronic Illn 3: 228–245. doi:10.1177/1742395307081502. PubMed: 18083679. 18083679

[5] Restrepo BI, Camerlin AJ, Rahbar MH, Wang W, Restrepo MA et al. (2011) Cross-sectional assessment reveals high diabetes prevalence among newly-diagnosed tuberculosis cases. Bull World Health Organ 89: 352–359. doi:10.2471/BLT.10.085738. PubMed: 21556303. 21556303 PMC3089389

[6] Dooley KE, Tang T, Golub JE, Dorman SE, Cronin W (2009) Impact of diabetes mellitus on treatment outcomes of patients with active tuberculosis. Am J Trop Med Hyg 80: 634–639. PubMed: 19346391. 19346391 PMC2750857

[7] Alisjahbana B, Sahiratmadja E, Nelwan EJ, Purwa AM, Ahmad Y et al. (2007) The effect of type 2 diabetes mellitus on the presentation and treatment response of pulmonary tuberculosis. Clin Infect Dis 45: 428–435. doi:10.1086/519841. PubMed: 17638189. 17638189

[8] Cheng MH (2010) Asia-Pacific faces diabetes challenge. Lancet 375: 2207–2210. doi:10.1016/S0140-6736(10)61014-8. PubMed: 20626091. 20626091

[9] Wang Q, Han X, Ma A, Wang Y, Bygbjerg IC et al. (2012) Screening and intervention of diabetes mellitus in patients with pulmonary tuberculosis in poverty zones in China: rationale and study design. Diabetes Res Clin Pract 96: 385-391. doi:10.1016/j.diabres.2011.11.012. PubMed: 22153416. 22153416

[10] Schwenk A, Hodgson L, Rayner CF, Griffin GE, Macallan DC (2003) Leptin and energy metabolism in pulmonary tuberculosis. Am J Clin Nutr 77: 392–398. PubMed: 12540399. 12540399 10.1093/ajcn/77.2.392

[11] van Crevel R, Karyadi E, Netea MG, Verhoef H, Nelwan RH et al. (2002) Decreased plasma leptin concentrations in tuberculosis patients are associated with wasting and inflammation. J Clin Endocrinol Metab 87: 758–763. doi:10.1210/jc.87.2.758. PubMed: 11836317. 11836317

[12] Buyukoglan H, Gulmez I, Kelestimur F, Kart L, Oymak FS et al. (2007) Leptin levels in various manifestations of pulmonary tuberculosis. Mediat Inflamm:64859: 64859 PubMed: 17497033. 10.1155/2007/64859 PMC1804295 17497033

[13] Yüksel I, Sencan M, Dökmetaş HS, Dökmetaş I, Ataseven H et al. (2003) The relation between serum leptin levels and body fat mass in patients with active lung tuberculosis. Endocr Res 29: 257–264. doi:10.1081/ERC-120025033. PubMed: 14535627. 14535627

[14] Halaas JL, Gajiwala KS, Maffei M, Cohen SL, Chait BT et al. (1995) Weight-reducing effects of the plasma protein encoded by the obese gene. Science 269: 543–546. doi:10.1126/science.7624777. PubMed: 7624777. 7624777

[15] Leite-Moreira AF, Soares JB (2007) Physiological, pathological and potential therapeutic roles of ghrelin. Drug Discov Today 12: 276–288. doi:10.1016/j.drudis.2007.02.009. PubMed: 17395087. 17395087

[16] Lord GM, Matarese G, Howard JK, Baker RJ, Bloom SR et al. (1998) Leptin modulates the T-cell immune response and reverses starvation-induced immunosuppression. Nature 394: 897-901. doi:10.1038/29795. PubMed: 9732873. 9732873

[17] Cakir B, Yönem A, Güler S, Odabaşi E, Demirbaş B et al. (1999) Relation of leptin and tumor necrosis factor alpha to body weight changes in patients with pulmonary tuberculosis. Horm Res 52: 279–283. doi:10.1159/000023495. PubMed: 10965207. 10965207

[18] Yurt S, Erman H, Korkmaz GG, Kosar AF, Uysal P et al. (2013) The role of feed regulating peptides on weight loss in patients with pulmonary tuberculosis. Clin Biochem 46: 40-44. doi:10.1016/j.clinbiochem.2012.09.008. PubMed: 23000316. 23000316

[19] Kim JH, Lee CT, Yoon HI, Song J, Shin WG et al. (2010) Relation of ghrelin, leptin and inflammatory markers to nutritional status in active pulmonary tuberculosis. Clin Nutr 29: 512-518. doi:10.1016/j.clnu.2010.01.008. PubMed: 20153570. 20153570

[20] Chang SW, Pan WS, Lozano Beltran D, Oleyda Baldelomar L, Solano MA et al. (2013) Gut hormones, appetite suppression and cachexia in patients with pulmonary TB. PLOS ONE 8: e54564. doi:10.1371/journal.pone.0054564. PubMed: 23358528. 23358528 PMC3554726

[21] Schwenk A, Macallan DC (2000) Tuberculosis, malnutrition and wasting. Curr Opin Clin Nutr Metab Care 3: 285–291. doi:10.1097/00075197-200007000-00008. PubMed: 10929675. 10929675

[22] Karyadi E, Dolmans WM, West CE, Van Crevel R, Nelwan RH et al. (2007) Cytokines related to nutritional status in patients with untreated pulmonary tuberculosis in Indonesia. Asia Pac J Clin Nutr 16: 218-226. PubMed: 17468076. 17468076

[23] Unsal E, Aksaray S, Köksal D, Sipit T (2005) Potential role of interleukin 6 in reactive thrombocytosis and acute phase response in pulmonary tuberculosis. Postgrad Med J 81: 604-607. doi:10.1136/pgmj.2004.030544. PubMed: 16143693. 16143693 PMC1743352

[24] Miller SG, De Vos P, Guerre-Millo M, Wong K, Hermann T et al. (1996) The adipocyte specific transcription factor C-EBPalpha modulates human ob gene expression. Proc Natl Acad Sci U_S_A 93: 5507–5511. doi:10.1073/pnas.93.11.5507. PubMed: 8643605. 8643605 PMC39276

[25] Bottasso O, Bay ML, Besedovsky H, del Rey A (2007) The immuno-endocrine component in the pathogenesis of tuberculosis. Scand J Immunol 66: 166–175. doi:10.1111/j.1365-3083.2007.01962.x. PubMed: 17635794. 17635794

[26] Beutler B, Cerami A (1987) Cachectin: More than a tumor necrosis factor. N Engl J Med 316: 379–385. doi:10.1056/NEJM198702123160705. PubMed: 3543677. 3543677

[27] Fujiwara H, Kleinhenz ME, Wallis RS, Ellner JJ (1986) Increased interleukin-1 production and monocyte suppressor cell activity associated with human tuberculosis. Am Rev Respir Dis 133: 73–77. PubMed: 2935057. 2935057 10.1164/arrd.1986.133.1.73

[28] Langhans W (2000) Anorexia of infection: current prospects. Nutrition 16: 996-1005. doi:10.1016/S0899-9007(00)00421-4. PubMed: 11054606. 11054606

[29] Marzullo P, Verti B, Savia G, Walker GE, Guzzaloni G et al. (2004) The relationship between active ghrelin levels and human obesity involves alterations in resting energy expenditure. J Clin Endocrinol Metab 89: 936-939. doi:10.1210/jc.2003-031328. PubMed: 14764817. 14764817

[30] Espelund U, Hansen TK, Ørskov H, Frystyk J (2003) Assessment of ghrelin. APMIS 109: 140–145. PubMed: 12874966. 12874966

[31] Suchanek P, Kralova Lesna I, Mengerova O, Mrazkova J, Lanska V et al. (2012) Which index best correlates with body fat mass: BAI, BMI, waist or WHR? Neuro Endocrinol Lett 33: 78-82. PubMed: 23183515. 23183515

[32] Gallagher D, Heymsfield SB, Heo M, Jebb SA, Murgatroyd PR et al. (2000) Healthy percentage body fat ranges: an approach for developing guidelines based on body mass index. Am J Clin Nutr 72: 694-701. PubMed: 10966886. 10966886 10.1093/ajcn/72.3.694

[33] Ma GS, Li YP, Hu XQ, Cui ZH, Yang XG et al. (2006) Report on childhood obesity in China (2). Verification of BMI classification reference for overweight and obesity in Chinese children and adolescents. Biomed Environ Sci 19: 1-7. 16673811

[34] Du SM, Ma GS, Li YP, Fang HY, Hu XQ et al. (2010) Relationship of body mass index, waist circumference and cardiovascular risk factors in Chinese adult. Biomed Environ Sci 23: 92-101. PubMed: 20514983. 20514983 10.1016/S0895-3988(10)60037-2

[35] Zhang ZQ, Deng J, He LP, Ling WH, Su YX et al. (2013) Comparison of various anthropometric and body fat indices in identifying cardiometabolic disturbances in chinese men and women. PLOS ONE 12; 8: e70893. 10.1371/journal.pone.0070893 PMC3741370 23951031

[36] Mohan VP, Scanga CA, Yu K, Scott HM, Tanaka KE, Tsang E et al. (2001) Effects of tumornecrosis factor alpha on host immune response in chronic persistent tuberculosis: possible role for limiting pathology. Infect Immun 69: 1847–1855. doi:10.1128/IAI.69.3.1847-1855.2001. PubMed: 11179363. 11179363 PMC98092

[37] Goyal R, Faizy AF, Siddiqui SS, Singhai M (2012) Evaluation of TNF-α and IL-6 Levels in Obese and Non-obese Diabetics: Pre- and Postinsulin Effects. N Am. J Med Sci 4: 180-184. 10.4103/1947-2714.94944 PMC3334258 22536561

[38] Douvas GS, Looker DL, Vatter AE, Crowle AJ (1985) Gamma interferon activates human macrophages to become tumoricidal and leishmanicidal but enhances replication of macrophage-associated mycobacteria. Infect Immun 50: 1-8. PubMed: 3930401. 3930401 10.1128/iai.50.1.1-8.1985 PMC262123

[39] Sarraf P, Frederich RC, Turner EM, Ma G, Jaskowiak NT et al. (1997) Multiple cytokines and acute inflammation raise mouse leptin levels: potential role in inflammatory anorexia. J Exp Med 185: 171–175. doi:10.1084/jem.185.1.171. PubMed: 8996253. 8996253 PMC2196098

[40] German JP, Wisse BE, Thaler JP, Oh IS, Sarruf DA et al. (2010) Leptin deficiency causes insulin resistance induced by uncontrolled diabetes. Diabetes 59: 1626–1634. doi:10.2337/db09-1918. PubMed: 20424233. 20424233 PMC2889761

[41] Marino JS, Xu Y, Hill JW (2011) Central insulin and leptin-mediated autonomic control of glucose homeostasis. Trends Endocrinol Metab 22: 275–285. PubMed: 21489811. 21489811 10.1016/j.tem.2011.03.001 PMC5154334

[42] van Lettow M, van der Meer JW, West CE, van Crevel R, Semba RD (2005) Interleukin-6 and human immunodeficiency virus load, but not plasma leptin concentration, predict anorexia and wasting in adults with pulmonary tuberculosis in Malawi. J Clin Endocrinol Metab 90: 4771-4776. doi:10.1210/jc.2004-2539. PubMed: 15928249. 15928249

